# One-pot construction of nitrogen-rich polymeric ionic porous networks for effective CO_2_ capture and fixation†

**DOI:** 10.1039/d1py01121a

**Published:** 2021-11-24

**Authors:** Chenxiang Ai, Xinquan Ke, Juntao Tang, Xincun Tang, Raed Abu-Reziq, Jian Chang, Jinyin Yuan, Guipeng Yu, Chunyue Pan

**Affiliations:** College of Chemistry and Chemical Engineering, Central South University Changsha 410083 China gilbertyu@csu.edu.cn panchunyue@csu.edu.cn; Institute of Chemistry, Casali Center of Applied Chemistry, The Center for Nanoscience and Nanotechnology, The Hebrew University of Jerusalem Jerusalem 91904 Israel; Department of Materials and Environmental Chemistry, Stockholm University Stockholm 10691 Sweden jiayin.yuan@mmk.su.se

## Abstract

Facile preparation of ionic porous networks (IPNs) with large and permanent porosity is highly desirable for CO_2_ capture and transformation but remains a challenge. Here we report a one-pot base-mediated construction of nitrogen-rich IPNs through a combination of nucleophilic substitution and quaternisation chemistry from H-imidazole. This strategy, as proven by the model reactions of 1*H*-imidazole or 1-methyl-1*H*-imidazole with cyanuric chloride, allows for fine regulation of porosity and physicochemical properties, leading to nitrogen-rich IPNs featuring abundant ionic units and radicals. The as-prepared networks, termed IPN-CSUs, efficiently capture CO_2_ (80.1 cc g^−1^ at 273 K/1 bar) with an ideal CO_2_/N_2_ selectivity of 139.7. They can also effectively catalyse the cycloaddition reaction between CO_2_ and epoxides with high yields of up to 99% under mild conditions (0.1 MPa, 298 K), suggesting their possible applications in the fields of both selective molecular separation and conversion. Unlike the previously known strategies generally involving single coupling chemistry, our strategy combining two coupling routes in one pot appears to be unique and potentially applicable to other building blocks.

## Abstract 2

A scale-up strategy through combing nucleophilic substitution and quaternisation chemistry was developed to build highly porous ionic networks with high CO_2_ storage capacity and high catalytic efficiency for CO_2_ conversion even under 1800 ppm CO_2_.

## Graphical abstract

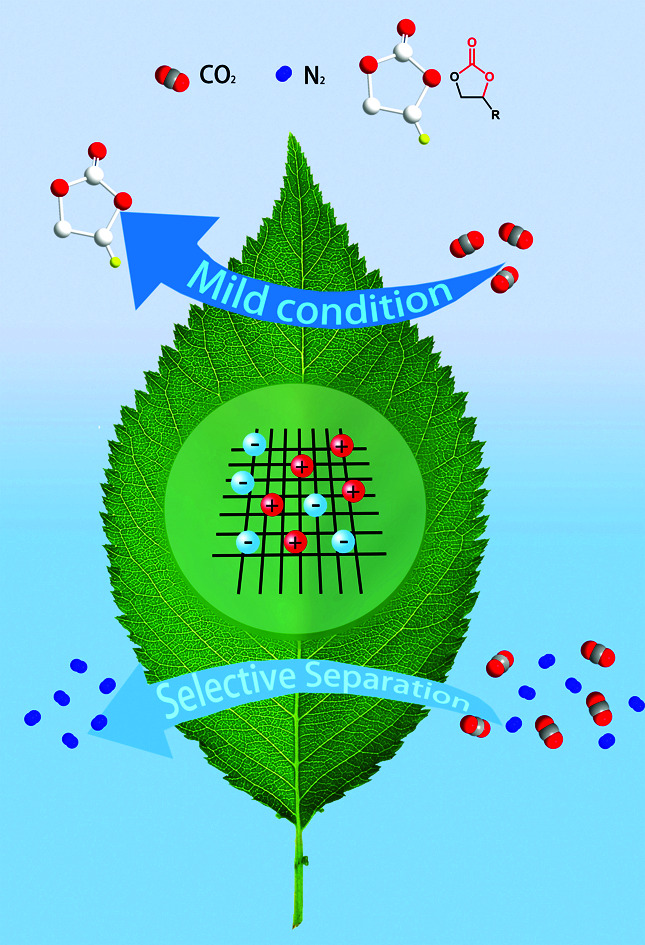

## 1. Introduction

The capture of carbon dioxide (CO_2_) from fossil-fueled power plants is of great significance, considering the deteriorating climate change and global warming. Meanwhile, the ever-increasing energy demand coupled with the lack of mature technologies for the widespread use of renewable energy still marks fossil fuels as a primary energy source. This points to the fact that the development of CO_2_ as a C1 resource is rather important to satisfy the ever-growing energy demand.^1,2^ A two-in-one technique that can selectively capture CO_2_ and efficiently convert it into value-added products at the same time would be highly desirable.

Ionic porous networks (termed IPNs) represent amorphous or crystalline porous polymers with evenly distributed ionic units and well-defined nanopores.^3^ These endow them with high affinity towards guest molecules through electrostatic interaction, in contrast to ion-free porous materials either from inorganic or organic components. Unlike their electronically neutral counterparts, IPNs allow easy functionalization through incorporating targeted cations or anions into the polymer chain or through simple ion exchange for task-specific applications.^4–6^ With a high charge density, good porosity and synthetic diversity, IPNs have recently emerged as a new platform for gas storage,^7^ catalysis,^8^ guest sensing^9^ and chemical separation.^10^ From the chemistry point of view, employing different ionic moieties into the polymer backbone through direct polymerization is the most common approach for constructing IPNs.^11^ Various synthesis strategies including coupling reactions (*i.e.*, Sonogashira reaction,^12^ Suzuki–Miyaura cross-coupling reaction^13^ and Yamamoto reaction^14^), Friedel–Crafts reaction,^15^ free radical polymerization,^16^ trimerization reaction,^17^ Schiff-base reaction^18^ and more have been successfully applied. For achieving a high CO_2_ capture and storage ability from gas mixtures through IPNs, several key design requirements are synergistically considered, *i.e.* pore parameters, host–guest interactions and charge effect. For example, IPNs with vinyl phosphonium or vinylimidazolium salts through free radical polymerization have exhibited acceptable Brunauer–Emmett–Teller (BET) surface areas (up to 758 m^2^ g^−1^) and high CO_2_ adsorption capacity (2.23 mmol g^−1^ at 273 K).^19^ Using a similar method, Luo *et al.* reported a series of ionic-based metallosalen-containing IMOPs with a surface area of 590 m^2^ g^−1^.^20^ Ionic covalent organic frameworks (COFs) feature 3-fold interpenetrated structures with a diamond (dia) topology, namely 3D-ionic-COF-1, which could exhibit significantly improved porosity with a specific surface area of 966 m^2^ g^−1^, due to its open and ordered pore structure.^21^ The highest BET surface area (1532 m^2^ g^−1^) for IPNs was demonstrated by PyTTA-BFBIm-iCOF which was built through the regulation of ionic interfaces that are well aligned yet spatially confined on the pore walls.^22^ However, for achieving high porosity, rigid and contortive building blocks or linkages are generally required in these systems and the ionic content is rather low relative to the overall polymer architecture. The obtainment of a large surface area and pore volume of the IPNs combined with a high ionic density remains far more challenging than that for their neutral counterparts due to the strong electrostatic repulsion. Besides, IPNs still face challenges concerning their tedious synthesis procedure as well as production cost and efficiency.^23–25^ Therefore, designing and synthesizing IPNs with a satisfactory surface area in a facile and scalable manner are of great significance.

Bearing this in mind, herein we disclose a facile one-pot construction strategy for building highly porous ionic networks through a combination of nucleophilic substitution and quaternization chemistry starting from cost-effective commercial monomers, *i.e.* 1*H*-imidazole (H-Imz) and cyanuric chloride (CC). To assess the feasibility of this competition chemistry, we first probe two model reactions between H-Imz or 1-methyl-1*H*-imidazole and CC. This strategy appears to be unique, allowing good control over the physicochemical properties and porous structure, leading to networks (IPN-CSUs) with an ultra-high nitrogen content (over 33 wt%) and ionic units and radicals in the chain. The as-prepared IPNs with polar group-modified surfaces can enhance the dipole–quadrupole interactions between frameworks and guest CO_2_ molecules. They can selectively capture CO_2_ from CO_2_/N_2_ mixtures, as demonstrated by the high-yield cycloaddition reactions (up to 99%) of epoxides with CO_2_ to cyclic carbonates under mild conditions (0.1 MPa, 298 K). A wide range of epoxides can be effectively formed with excellent yields by cycloaddition over the IPN-CSU catalysts. This work opens up a new pathway to build high performance sorbents and catalysts for the advancement of capture and utilization of CO_2_.

## 2. Experimental section

### 2.1 Model reaction between 1*H*-imidazole and cyanuric chloride

To a 100 mL three-necked round-bottomed flask equipped with a stirrer, a nitrogen inlet, and a Dean–Stark trap fitted with a condenser, 40 mmol of H-Imz was charged and dissolved in 25 mL of tetrahydrofuran to give a clear solution. 22 mmol of anhydrous potassium carbonate was added into the solution under constant stirring, and then 10 mL of toluene was added. The mixture was heated to reflux for 2 h, and the water produced was azeotroped off with toluene. After being cooled to room temperature, CC (8 mmol) which was dissolved in tetrahydrofuran, was added slowly into the H-Imz solution in 2 h. The mixture was kept in a glass vessel at freezing temperature (273 K) for another 8 h. The obtained product was filtered, rinsed with water and then the filter cake was vacuum-dried at 80 °C for 12 h (yield: 95%). ^1^H NMR (400 MHz, DMSO-d_6_): *δ* 9.08 (s, 1H), 8.31 (t, *J* = 1.4 Hz, 1H), 7.22 (d, *J* = 1.6 Hz, 1H). ^13^C NMR (100 MHz, DMSO-d_6_): *δ* 162.11, 137.52, 131.31, 117.22. GC-TOF/Mass (C_12_H_9_N_9_): calculated: 279.0981; found: 279.0875.

### 2.2 Model reaction between 1-methyl-1*H*-imidazole and cyanuric chloride

30 mmol of 1-methyl-1*H*-imidazole (Me-Imz) and 8 mmol cyanuric chloride were charged into a dried three-necked flask protected by nitrogen and then 50 mL anhydrous DMF was added by injection. After mixing evenly, the reaction mixture was maintained at 0 °C for 6 h. Then it was recovered to room temperature and further heated to 80 °C for another 8 h. After cooling to room temperature, the precipitate was collected and then immersed in anhydrous methanol. It was rinsed with 30 mL tetrahydrofuran and 50 mL dichloromethane, and then dried in an oven at 80 °C for 12 h. A pale solid was obtained with a yield of 91%.

### 2.3 General synthesis route to ionic porous networks (IPN-CSUs)

A typical polymerization procedure to form IPN-CSU23 is given as an example. To a sealed glass vessel a mixture of H-Imz (30 mmol) and anhydrous potassium carbonate (K_2_CO_3_ 18 mmol) was charged, and 20 mL anhydrous *N*-methylpyrrolidone and 30 mL toluene were added later. They were mixed evenly by stirring for 0.5 h, and then heated to 120 °C for 4 h to promote dehydration under the protection of nitrogen. Then the mixture was cooled down to room temperature, and cyanuric chloride was added in three or four batches. The mixture was reheated and then the temperature was maintained at 50 °C for 2 h, 75 °C for 4 h, and 80 °C for 6 h. After cooling to room temperature, the obtained precipitate was settled in anhydrous methanol, and the product was obtained by filtration. The solids soaked in ethanol were extracted with dichloromethane and acetone overnight and 2 h, respectively, and the product with a yield of 82% was obtained after vacuum drying.

## 3. Results and discussion

### 3.1 Preparation and primary properties of IPN-CSUs

**Scheme 1:**
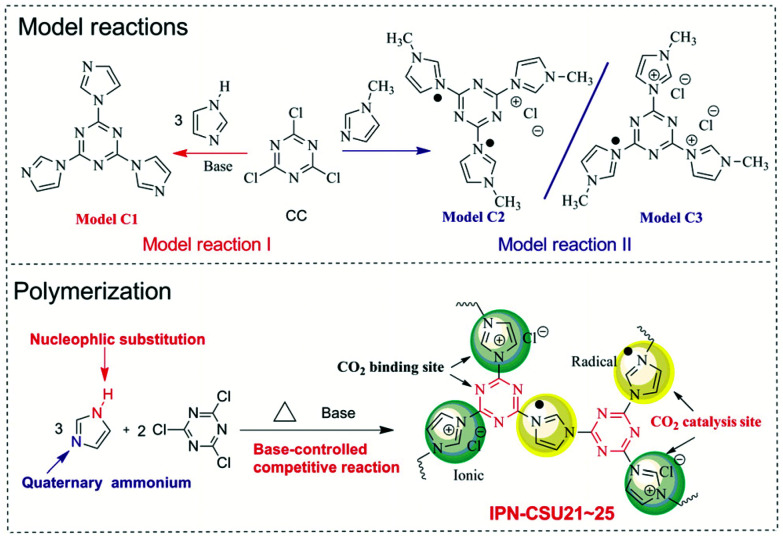
Model reactions (top) and the synthesis route of IPN-CSU polymers (down).

To probe the feasibility of this polymerization, two model reactions between 1*H*-imidazole (H-Imz) or its derivative and cyanuric chloride (CC) were conducted. Their general synthesis routes are shown in Scheme 1. The nucleophilic displacement reaction of H-Imz and CC was completed in the presence of a base catalyst like anhydrous potassium carbonate. The N–H of phenol-like H-Imz, which is acidic, would be converted to its more reactive form, an aza-nitrogen anion, in the presence of appropriate weak bases, demonstrating high activity in the reaction. This transformation was promoted in the presence of toluene to allow azeotropic distillation of the water formed as a by-product. Further displacement reaction with halogenated compounds, *i.e.* CC, afforded the target triimidazole-1,3,5-triazine (Model C1) quantitatively. Considering the high activity of CC, another weaker base, *e.g. N*,*N*′-diisopropylethylamine or potassium bicarbonate as the catalyst also afforded the target model compound smoothly with a yield of over 90%. This proves the success of the nucleophilic substitution chemistry, possibly due to the highly acidic nature of N–H in H-Imz. Note that with an unsatisfactory base catalyst in this reaction the yield of the target Model C1 decreased significantly. By inference, another competitive route, *i.e.* quaternization of imidazole would also occur considering the basic nature of 3-N in H-Imz and the high activity of CC. Further evidence was provided by the model reaction between 1-methyl-1*H*-imidazole (Me-Imz) and cyanuric chloride. It proceeds efficiently in polar solvents like acetone, dimethylformamide (DMF) or THF without the presence of any catalyst. Since N–H of Me-Imz was replaced by the methyl substituent, only quaternization was allowed (Scheme 1). Interestingly, a mixture containing Model C2 and C3 rather than a single product was obtained in this reaction, although we have tried to purify the products using column chromatography several times.

The chemical structure of Model-C1 was confirmed by proton nuclear magnetic resonance (^1^H-NMR) spectroscopy, carbon nuclear magnetic resonance (^13^C-NMR) spectroscopy and gas chromatography high-throughput time-of-flight mass spectrometry (GC/TOF-MS). As shown in the ^1^H-NMR spectrum of Model-C1 (ESI, Fig. S1†), the N–H signal of H-Imz completely disappeared, and the ratio and position of H_1_, H_2_, and H_3_ protons are well consistent with the target compound. The ^13^C-NMR spectrum also proves its chemical structure (ESI, Fig. S2†). The content of chloride ion was further determined quantitatively by anion exchange experiment (AEC, chloride form, ESI S3†). Here, AEC (Cl^−^) represents the amount of chloride ion per mass of the sample. For Model C1, the AEC value is found to be 0 mmol g^−1^, confirming that there no quarternisation occurred in this reaction when excess base catalyst was charged. For the mixture of Model C2 and C3, the AEC value is recorded to be 3.4 mmol g^−1^, only half of the theoretical value (6.9 mmol g^−1^) of our proposed triazine-triimidazolium salt. It would be reasonable, considering the strong electron-pulling effect of the 1,3,5-triazine-based conjugated network which forces the reduction of about 50% of triazinium chloride to a radical form stabilized by the conjugated π system.^26^ These model reactions proved that H-Imz would act as a bifunctional monomer (A-A type) under the selected reaction conditions. More importantly, two coupling chemistries, *i.e.*, nucleophilic substitution and quarternisation truly existed. This method allows us to tune the composition of the obtained polymer by varying the ratio between the monomer and the catalyst, thus modulating its physicochemical properties.

Based on the preliminary results from these model reactions, we tried to probe a feasible polymerization route to obtain highly crosslinked IPNs (Table 1). Preliminary screening and optimization of catalyst types, catalyst concentrations, and solvents aimed to generate high-surface-area networks in high yields. The crosslinked products can be successfully obtained by using potassium carbonate, potassium bicarbonate or alkylamine as the catalyst. With the charge 0.55 times that of an equivalent potassium carbonate catalyst relative to H-Imz, the yield of the IPN-CSU21 polymer is up to 91%, while that of IPN-CSU23 is slightly lower (82%), which may be due to the side reactions of the imidazole potassium salt at high temperatures during the removal of toluene. Our results show that the reactions which used azeotropic distillation to promote the formation of salt, and used a solvent mixture rather than a single solvent, are more favourable for obtaining IPNs with high specific surface areas. The solvent mixture is conducive to preventing the premature precipitation of oligomers or intermediates with a low crosslinking degree produced in the reaction process, so as to finally improve the crosslinking degree of the product.

**Table 1:** The preparation and basic properties of IPN-CSUs

Sample	Cat	Cat content^a^ (mol mol^−1^)	Temp. (°C)	Solvent	*S*_BET_ b (m^2^ g^−1^)	Yield (wt%)
IPN-CSU21	K_2_CO_3_	0.55 : 1	80	NMP	53	91
IPN-CSU22	K_2_CO_3_ c	0.55 : 1	80	NMP	92	89
IPN-CSU23	K_2_CO_3_ c	0.6 : 1	80	NMP/THF	210	82
IPN-CSU24	KHCO_3_ c	1.2 : 1	80	NMP/THF	169	90
IPN-CSU25	DIPEA d	1.2 : 1	80	THF	11	88

a The molar number of the catalyst relative to H-Imz.

b Brunauer–Emmett–Teller specific surface area.

c Toluene: azeotropic solvents for the distillation of water.

d DIPEA: *N*,*N*′-diisopropylethylamine.

The obtained IPN-CSU polymers are light yellow or yellow powders, while the colour of the polymer samples obtained by azeotropic distillation is slightly darker (ESI, Fig. S3†). The solubility test showed that all products were insoluble in common organic solvents such as in methanol and acetone, and also in high polarity solvents such as NMP, DMAC and DMF even at elevated temperatures. With simple moulding, the polymerization product can be pressed into a well-defined stable shape as desired. A wafer with a diameter of about 2 cm for IPN-CSU23 can be obtained (ESI, Fig. S3†). Elemental analysis demonstrated that the ratio of nitrogen to carbon (ESI, Table S1†) was close to the theoretical ratio of the feed monomer, indicating that almost all monomers participated in the reaction according to our procedures. The AEC (Cl^−^) of a typical network IPN-CSU23 is estimated to be 3.3 mmol g^−1^, close to that of the mixtures obtained in the model reaction II. The polymerization possibly followed a similar reaction mechanism with the two model reactions, in which nucleophilic displacement occurred and at the same time imidazolium chloride units obtained by quaternization reaction were reduced by their own Cl^−^ to a neutral radical state. It is proposed that there are two possible routes for H-Imz to generate IPN-CSUs (ESI, Fig. S4†). For the first route, H-Imz is substituted by CC to give intermediate I, while the remaining underwent quaternary ammonium ionization to afford intermediate II (Route 2). After that, further coupling reactions between intermediate I and H-Imz or intermediate II occurred, producing a crosslinked network with a neutral radical state. This facile strategy combining two coupling routes is different from those known strategies which generally involve only single coupling chemistry, and our strategy would be applicable to other building blocks, such as 4*H*-1,2,4-triazole or 1*H*-1,2,3-triazole. Note that the proposed structure was different from that of SPIN-1 starting from 2,4,6-tri(1*H*-imidazol-1-yl)-1,3,5-triazine and CC.^27^ The SPIN-1 network was obtained by the quaternization of triimidazole triazine with CC followed by hydrolysis and *in situ* assembly. More specifically, the preparation of such ionic networks involves the hydrolysis of positively charged triazine, the release of negative/positive charges, and also the assembly of negative/positive charges *via* electrostatic interaction. The control of this polymerization is the key which determines the chemical connection and structure of the as-prepared polymeric networks. Under different polymerization conditions like temperature and catalyst nature, ionic polymeric networks with different chemical structures would be obtained.

**Fig. 1:**
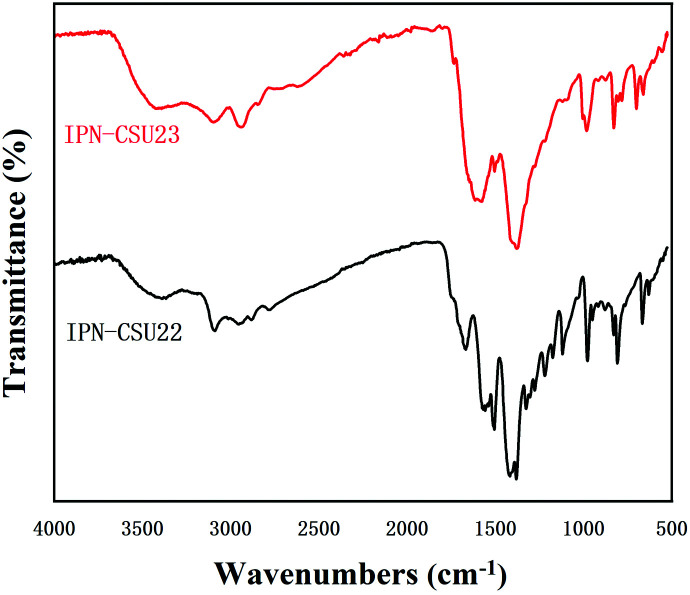
FT-IR spectra of IPN-CSU22 and IPN-CSU23.

**Fig. 2:**
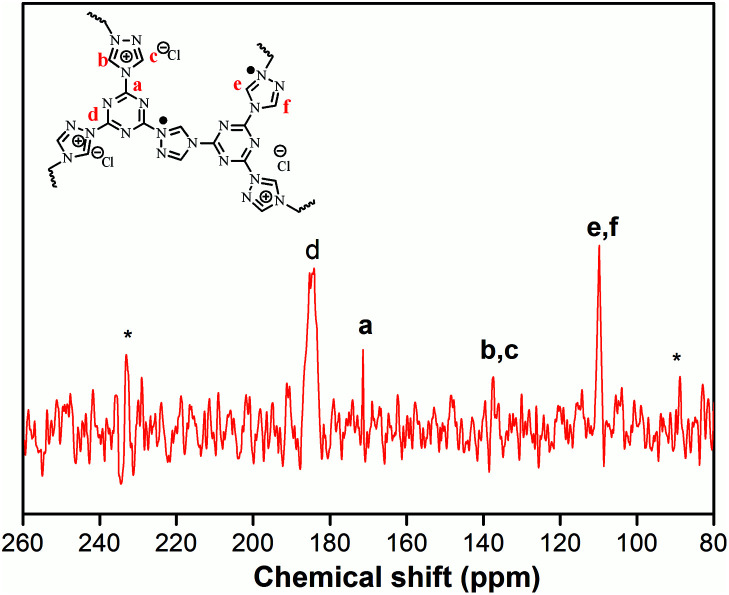
Solid-state ^13^C CP/MAS NMR spectrum of IPN-CSU22.

The structural integrity of our IPN-CSUs was characterized by Fourier transform infrared (FT-IR) (Fig. 1) and solid-state ^13^C cross polarization magic angle spinning (^13^C CP/MAS) NMR spectroscopic measurements (Fig. 2). The presence of an intense band at 1590 cm^−1^ (1500 cm^−1^) and 1326 cm^−1^ for C=N or C–N stretching in the triazine ring, as well as the disappearance of the band at 850 cm^−1^ for C–Cl out-of-plane vibration verifies the completion of polymerization. The signals in the solid-state ^13^C CP/MAS NMR spectrum of typical IPN-CSU22 are well consistent with the proposed polymer structure. In addition, our analysis of the FTIR spectra verified that the cross-linking degree and ionic unit content of the IPN-CSU polymer can be adjusted by changing the polymerization reaction conditions. The elemental content analysis of IPN-CSU22 and IPN-CSU23 polymers in the CHN mode further confirmed that half of the chlorine was ionized, and another half remained unchanged (ESI, Table S1†). Note that this route was different from that disclosed by Huang *et al.* We admit that these networks are starting from the same starting material while the chemical structures of the resulting networks are totally different due to different mechanisms. Our model reaction and spectroscopic measurements, as well as AEC experimental results confirm the different reaction routes. The key is to ensure ambient polymerization conditions to avoid the hydrolysis of CC.

The advantage of the IPN-CSU network as a recycled adsorbent and/or catalyst is based on its structural stability. Thermogravimetric analysis (TGA) proved their good stability against thermal decomposition (ESI, Fig. S5†). For instance, IPN-CSU23 retained 95% of its original weight even at 405 °C under a nitrogen atmosphere. IPN-CSU was found to be hydrophilic due to its high quarternisation degree. The broad featureless patterns in powder X-ray diffraction (PXRD) of IPN-CSU22 and IPN-CSU23 suggest their amorphous nature (ESI, Fig. S6†), possibly due to the thermodynamically irreversible coupling reaction.

**Fig. 3:**
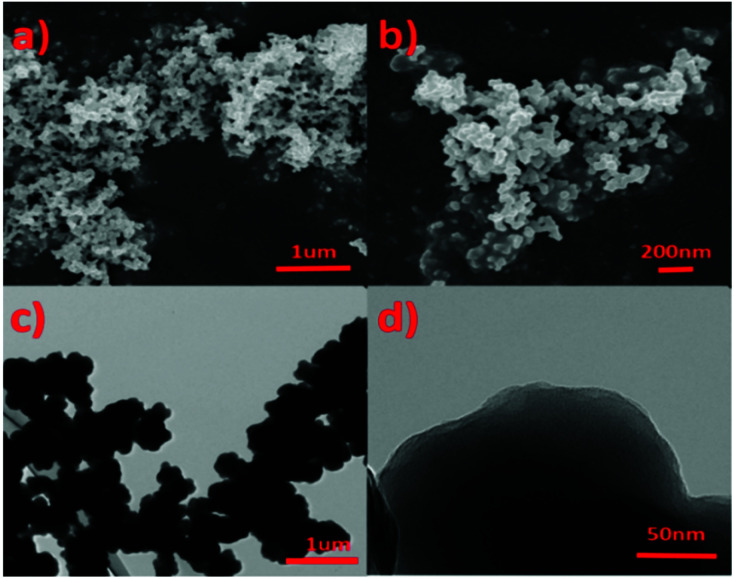
SEM (a) and TEM images (b) of IPN-CSU22.

Scanning electron microscopy (SEM) and transmission electron microscopy (TEM) were used to characterize the micro morphology of IPN-CSUs (Fig. 3 and Fig. S7, S8†). Scanning electron microscopy (SEM) analysis shows irregular aggregates built from primary particles 200–400 nm in size (Fig. 3a and b). The size and morphology of the microspheres displayed in the transmission electron microscopy (TEM, Fig. 3c) images are basically similar to those observed in the SEM images. The microsphere consists of abundant porous structures for IPN-CSU23, as observed in the high-resolution TEM images (Fig. 3d). The formation of the sphere-like morphology of the polymer may be ascribed to the minimized surface energy and the self-assembly of polymer chains.^28^

**Fig. 4:**
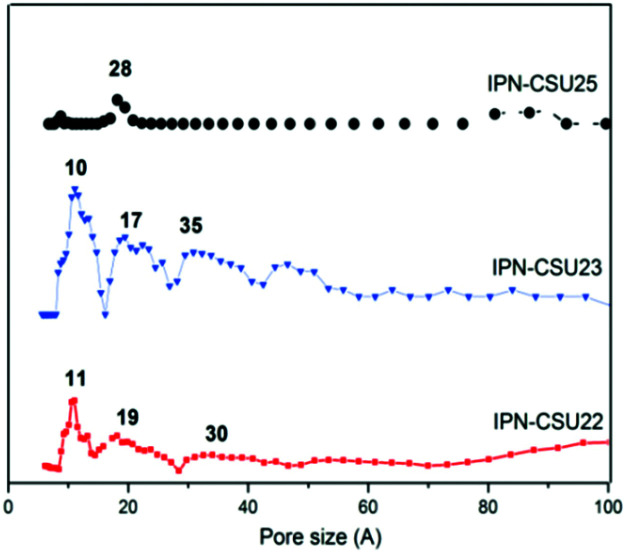
N_2_ sorption isotherms at 77 K for typical IPN-CSUs.

**Fig. 5:**
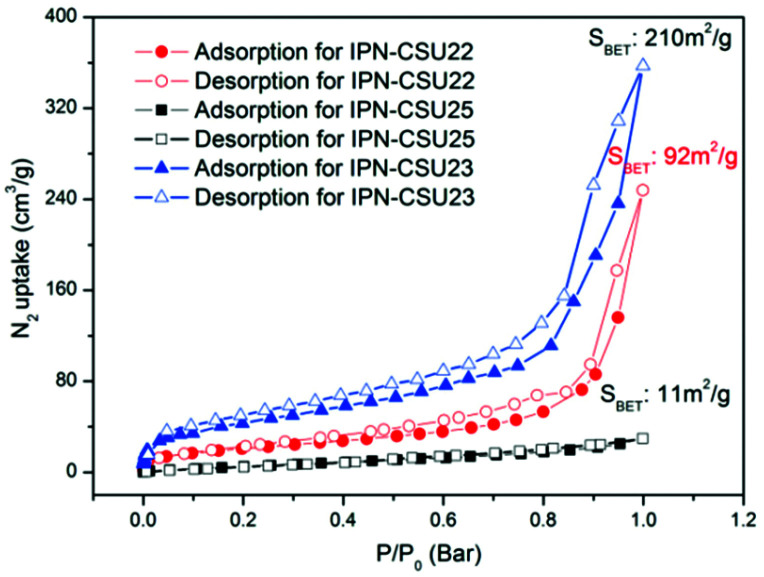
NL-DFT pore size distribution curves for typical IPN-CSUs.

Pore parameters of IPN-CSUs, including specific surface area, pore size, and pore volume are obtained by combining the adsorption–desorption isotherms (Fig. 4) with the Brunauer–Emmett–Teller (BET) equation and the nonlocal density functional theory (NL-DFT) model (Table 2). Nitrogen adsorption/desorption isotherms of IPF-CSU22 and IPN-CSU23 measured at 77 K show a certain gas uptake at a low relative pressure (*P*/*P*_0_ < 0.1), indicating the presence of micropores with a pore size of less than 2.0 nm. In the middle relative pressure range (*P*/*P*_0_ = 0.1–0.8), the adsorption curves increase slowly, suggesting the existence of some small mesopores. In the range above *P*/*P*_0_ = 0.8, the enhanced nitrogen uptake is indicative of large mesopores, and there are also macropores as the adsorption and desorption branches do not overlap below *P*/*P*_0_ = 1. These two polymers exhibit a complex adsorption behaviour consisting of a combination of type I and type III characters. IPN-CSU23 showed the highest BET surface area of 210 m^2^ g^−1^, which was comparable to that of SPIN-1, obtained from almost the same starting monomers. We had to admit the fact that the specific surface area and pore volume of IPF-CSUs are in general comparably lower than their non-ionic counterparts. It would be reasonable that in these nitrogen-rich IPN-CSUs with a high ionic content, the high interface-energy results in the collapse of the polymer pores through solvent removal by high-vacuum thermo-drying during BET analysis. Finally, the counter ions would occupy part of the pores, which makes them hard to detect by the N_2_ probe during the adsorption/desorption measurements. The pore size distribution is calculated based on the NL-DFT model (Fig. 5), and their curves are consistent with the adsorption–desorption isotherms. The results showed that its pore size distribution is related to the polymerization reaction conditions, where IPN-CSU22 and IPN-CSU23 demonstrate a high fraction of microporosity as well as certain mesoporosity. The total pore volume is 0.73 cm^3^ g^−1^ for IPN-CSU22, sufficiently higher than many known ionic porous polymers.^29–31^ The sample IPN-CSU23 after ion exchange with lithium bis(trifluoromethyl sulphonyl)imide (termed IPN-CSU23Tf) exhibited a slightly lower surface area and pore volume. This indicated that the porous properties would be easily regulated through a simple counter-ion exchange.

**Table 2:** Pore parameters and gas sorption capacity of IPN-CSUs

Entry	SSA^a^ (m^2^ g^−1^)	DPW^b^ (nm)	PV^c^ (cc g^−1^)	Cap^d^ (cc g^−1^)	*Q*_st_ e (kJ mol^−1^)	Sel^f^ (v/v)
IPN-CSU21	57	0.8, 1.8	0.18	26.7	28.3–30.2	60.4
IPN-CSU22	92	1.1, 1.9, 3.0	0.36	67.4	29.9–33.4	74.9
IPN-CSU23	210	1.0, 1.7, 3.5	0.45	80.1	29.4–37.8	126.2
IPN-CSU23Tfsi	184	0.7, 1.2	0.39	63.2	30.4–39.6	139.7
IPN-CSU24	153	1.4, 2.1	0.35	71.8	21.3–34.5	67.6
IPN-CSU25	31	2.8	0.09	12.9	26.5–30.6	45.1

a BET specific surface area (SSA).

b Dominant pore width (DPW).

c Pore volume (PV).

d CO_2_ capacity at 273 K and 1 bar (CAP).

e *Q*_st_: heat of adsorption at zero loading.

f Selectivity: initial ideal selectivity.

### 3.2 Selective capture performance of IPN-CSUs towards CO_2_

**Fig. 6:**
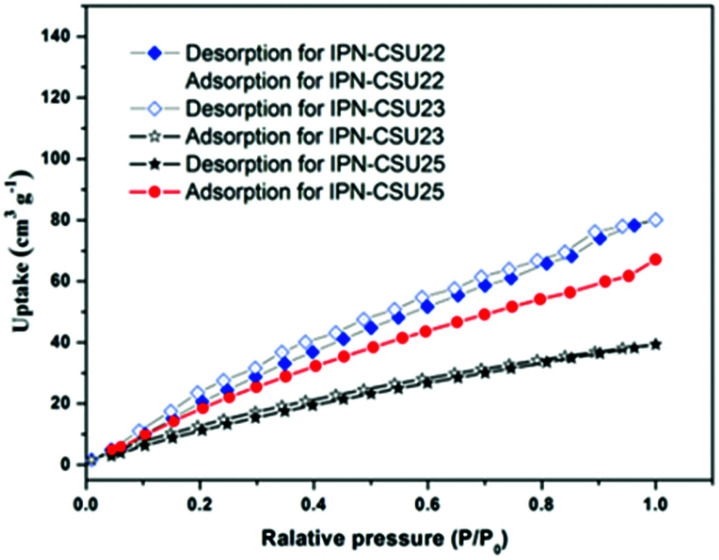
CO_2_ adsorption/desorption curves of IPN-CSUs at 273 K.

The capture and storage capacity of the as-prepared polymers towards CO_2_ was surveyed by gas adsorption and desorption experiments. Benefitting from the high content of nitrogen and ionic units, they exhibit high CO_2_ adsorption and desorption capabilities at low pressures (Fig. 6). IPN-CSU23 with the highest pore volume demonstrated the highest capacity of up to 80.1 cc g^−1^ towards CO_2_ at 273 K and 1 bar. This performance was significantly higher than SPIN-1 (24.7 cc g^−1^, 273 K/1 bar) and most known IPNs like IPN-CSU1 and PIP-Bn-Cl.^7,21^ More importantly, it was comparable to those of state-of-art highly porous neutral polymers like R/HMTA-0.42 (42.6 cc g^−1^) and TATHCP (64.1 cc g^−1^) under the same conditions (273 K/1 bar).^32,33^ The unprecedentedly high capacities of IPF-CSUs would be ascribed to the high-density distribution of ionic pairs and the presence of abundant N species in the pore surface. The IPN-CSU23-TFSI obtained after the anion exchange reaction is found to be more suitable for the kinetic capture of CO_2_ due to the high polarity of the fluorine element, therefore, it showed a strong interaction with CO_2_. The adsorption capacity of IPN-CSU23-TFSI remains at 63.2 cc g^−1^ at 273 K and 1 bar, despite its decreased pore volume in comparison with IPN-CSU23. These different CO_2_ adsorption properties can be interpreted by the influence of counter anions with different sizes and/or Lewis acidity. Note that the model compounds C1 and C2/C3 deliver almost negligible capacities for CO_2_ capture under the same conditions (ESI, Table S2†).

The isosteric heat of adsorption (*Q*_st_) plot of CO_2_ was calculated from the isotherms recorded at 273 K (Fig. 5) and 298 K (ESI, Fig. S9†) by using the Clausius–Clapeyron equation. Under a low load or close to zero load, the adsorption of CO_2_ by the adsorbent is close to single-layer adsorption, and a large number of ions on the surface of the adsorbent can adsorb and anchor the guest CO_2_ molecules through strong electrostatic induction. Due to the ultra-high nitrogen content on the polymer backbone, a large number of nitrogen-containing groups on the surface of IPN-CSUs can firmly immobilize guest CO_2_ molecules through dipole–dipole interaction. Therefore, for each IPN-CSU sample, the adsorption enthalpy of IPN-CSU for CO_2_ at low coverage is higher than 30 kJ mol^−1^. For example, IPN-CSU23 showed the strongest interaction with CO_2_ with an unprecedentedly high initial isosteric heat (*Q*_st_) of 37.8 kJ mol^−1^. After ion exchange, the sample IPN-CSU23-TFSI showed the highest initial *Q*_st_ of up to 39.6 kJ mol^−1^, due to the presence of bis(trifluoromethyl sulphonyl)imide counter anion with a stronger CO_2_ affinity than Cl^−^. Note that IPN-CSUs have high adsorption capacity, along with a good desorption capacity. The adsorption enthalpy of IPN-CSU is higher than most neutral porous materials such as CMP-1-COOH (33 kJ·mol^−1^).^34^ Referring to the strong CO_2_ affinity and high capacity, high-density distribution of ions and the presence of abundant N species, as well as the large pore volume, would play a crucial role here.

Given the high affinity for CO_2_, the selective capture of CO_2_ over N_2_ was probed using the simplified ideal adsorbed solution theory (IAST) model. The obtained nitrogen-rich IPN-CSU has a sufficiently high selectivity for CO_2_ than N_2_. Among the investigated samples, IPN-CSU23 gave the best ideal selectivity of up to 139.7, indicating that these networks may have industrial application prospects.

### 3.3 Catalytic conversion of epoxides with CO_2_ based on IPN-CSU catalysts

The IPN-CSUs with abundant ionic functionalities and N-rich species in their main chain feature strong adsorbate–ion interactions. This is beneficial for CO_2_ capture and appears to facilitate its transformation into value-added chemical feedstocks *e.g.* cyclic carbonates. Besides, counterion regulation *via* ion exchange reactions without altering the main chain would allow flexibility of structural variation and ensure high-efficiency of catalytic CO_2_ conversion.^5,35^ Many IPN-based catalysts have been found effective for conversion of CO_2_ to cyclic carbonates.^36–39^ For instance, Han *et al.* for the first time reported the employment of imidazolium containing IPNs for this catalytic experiment in 2007.^40^ Wang and co-workers found that ionothermally obtained meso-/macroporous PILs from bisvinylimidazolium salt precursors exhibited high activity toward efficient conversion of CO_2_ at atmospheric pressure and low temperature (70 °C).^41^ More recently, we reported an ionic porous polypyridinium (IPF-CSU-1) bearing high nitrogen content (20.3 wt%) that was capable of catalyzing conversion of CO_2_ into cyclic carbonates in a quantitative yield (>95%) favorably at room temperature (25 °C) and ambient pressure (0.1 MPa).^7^ It was found epoxide compounds can be efficiently activated by hydrogen bonding between the oxygen atom of epoxides and halide-rich IPN matrices, facilitating ring opening reactions by halides for carbonate production. However, most transformations of CO_2_ on porous catalysts was achieved under high CO_2_ pressures (3.0 MPa) and elevated temperatures (120 °C).^35–37^

Considering their high affinity towards CO_2_, IPN-CSUs are supposed to realize CO_2_ fixation under lower pressures close to real applications. We tried to investigate their efficiency for CO_2_ fixation and optimize the reaction conditions (Table 3). Our results demonstrated that the yields of 4-methyl-1,3-dioxolan-2-one on IPN-CSU22 and IPN-CSU23 under a pressure of 0.1 MPa at room temperature are up to 81% and 84%, respectively, in the absence of the auxiliary catalyst, *e.g.* tetrabutylammonium bromide (TBAB).

**Table 3:** Catalytic performance of IPN-CSU for CO_2_ cyclization

Entry	Catalyst	Time (h)	Pressure (MPa)	Temp. (°C)	Yield^a^ (%)
1	IPN-CSU22	48	0.1	25	81
2	IPN-CSU23	48	0.1	25	84
3	IPN-CSU22/TBAB	48	0.1	25	98
4	IPN-CSU23/TBAB	48	0.1	25	99
5	IPN-CSU22/TBAB	48	b	25	17
6	IPN-CSU23/TBAB	48	b	25	19
7	TBAB	48	0.1	25	45
8 ^35^	PCP-Cl	12	3	100	99
9 ^36^	POM3-IM	4	1	120	78
10 ^37^	F-PIL-Br	9	1	120	94
11 ^38^	[PPN]Cl	48	0.1	25	91
12 ^39^	Zn/HAzo-POP-1	48	0.1	25	99

a Determined by ^1^H NMR.

b CO_2_/N_2_ mixture, where CO_2_ partial pressure: 1800 ppm per 0.1 MPa.

With the coexistence of TBAB, the yield of the cycloaddition product of IPN-CSU23 increases to 99% under the same conditions. Interestingly, even under significantly lower pressure, like 1800 ppm CO_2_, the reaction catalysed by IPN-CSU23/TBAB also successfully affords 19% cyclic carbonates. A possible reaction mechanism for IPN-CSUs is shown in Fig. S10,† where the epoxides are activated by hydrogen bonding to the α-protons of the imidazolium ring, and the open-loop reaction occurs by the nucleophilic attack of epoxide by Cl^−^ to form an oxy ion intermediate. Then the intermediate reacts with CO_2_ to form the corresponding cyclic carbonates. Therefore, our metal-free IPN-CSUs feature remarkably high catalytic activities in the conversion of epoxides to the corresponding cyclic carbonates, and their performance was comparable to the previously reported best ionic porous polymers^37,38^ or the metal–porous polymer hybrid^39^ so far.

**Fig. 7:**
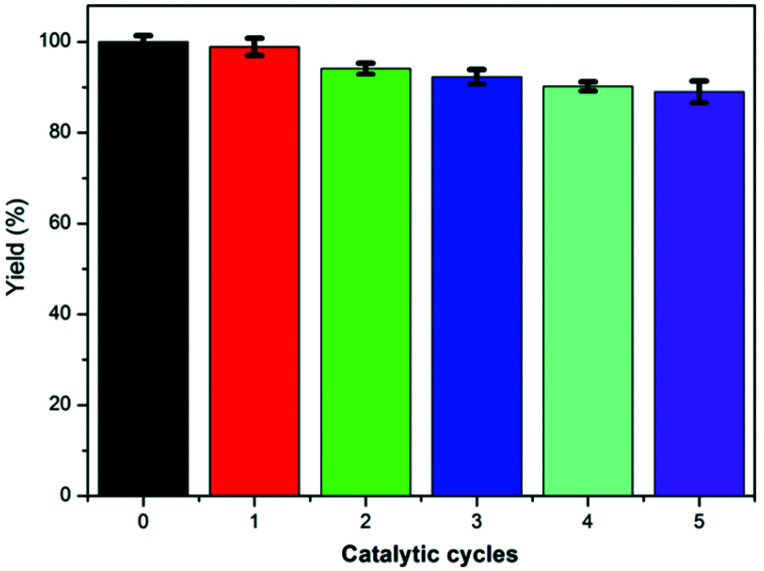
Recycling performance of the IPN-CSU23 catalyst for CO_2_ cyclization.

The reusability of our IPN-CSUs as catalysts was also investigated, and the reaction between CO_2_ and epibromohydrin catalysed by IPN-CSU23/TBAB was used as a model reaction. The catalysts were recovered through simple separation by filtration and recovery, and then reused directly for the next run. In each run, the high yield of the target product 4-methyl-1,3-dioxolan-2-one was retained and only a slight decrease was observed even after five straight runs (Fig. 7). From this point of view, the IPN-CSU possessed high structural stability, and also demonstrated attractive repeatability in the cycloaddition reaction of CO_2_. These results manifest the great potential of IPN-CSUs for CO_2_ capture and conversion into valuable products under mild conditions, thus utilizing CO_2_ as an environmentally friendly and renewable C1 source.

## 4. Conclusion

In summary, a series of ionic porous networks were prepared through a one-pot base-mediated competing reaction scheme of nucleophilic substitution and quaternization chemistry from H-Imz. This polymerization, as proven by the model reactions, would be completed under mild reaction conditions. The as-prepared IPN-CSUs showed good physicochemical stability, and exhibited a specific surface area of up to 210 m^2^ g^−1^ and a pore volume of 0.45 cc g^−1^. The primary properties and porous structure would be well tuned through a simple counter-anion exchange reaction. These nitrogen-rich networks with abundant ionic units and radicals in the chain showed strong affinity towards CO_2_. They demonstrated an unprecedentedly high adsorption capacity under low CO_2_ pressure as well as high selectivity. The good structural stability endows the IPN-CSUs with excellent heterogeneous catalytic activity for CO_2_ chemical fixation under mild ambient conditions. In particular, IPN-CSU23/TBAB has achieved 99% efficiency for conversion of carbon dioxide and propylene oxide at 25 °C and 0.1 MP, and retains an efficiency of 81% without TBAB, suggesting its great potential for industrial capture and utilization of CO_2_. The scalable synthesis method also provides new pathways for developing high performance IPNs.

## Conflicts of interest

There are no conflicts to declare.

## Supplementary Material

PY-013-D1PY01121A-s001
