# Supplementary material for: One-pot construction of nitrogen-rich polymeric ionic porous networks for effective CO2 capture and fixation
Source: Polym Chem. 2021 Nov 24;13(1):121–9. doi: 10.1039/d1py01121a (PMC8689585; doi:10.1039/d1py01121a)
Supplement: PY-013-D1PY01121A-s001 [file PY-013-D1PY01121A-s001.pdf]

Electronic Supplementary Material (ESI) for Polymer Chemistry.
This journal is © The Royal Society of Chemistry 2021

*Supporting Information*

# One-pot Construction of Nitrogen-rich Polymeric Ionic Porous Networks for Effective $CO_2$ capture and fixation

*Chenxiang Ai*[a], *Xinquan Ke*[a], *Juntao Tang*[a], *Xincun Tang*[a], *Raed Abu-Reziq*[b], *Jian Chang*[c], *Jinyin Yuan*[c], *, *Guipeng Yu*[a], *, *Chunyue Pan*[a], *

[a] *College of Chemistry and Chemical Engineering, Central South University, Changsha 410083, China*

[b] *Institute of Chemistry, Casali Center of Applied Chemistry, The Center for Nanoscience and Nanotechnology, The Hebrew University of Jerusalem, Jerusalem 91904, Israel*

[c] *Department of Materials and Environmental Chemistry, Stockholm University, Stockholm 10691, Sweden*

**CONTENTS**

**Section S1.** Materials

**Section S2.** Instruments and methods

**Section S3.** AEC measurements of model products and IPNs

**Section S4.** Determination for isosteric heat of adsorption

**Section S5.** Figures and Tables

**Scheme S1** Synthetic route of Model C1

**Scheme S2** Synthetic route of Model C2 and Model C3

**Figure S1** $^{1}H$ NMR spectrum for Model C1

**Figure S2** $^{13}C$ NMR spectrum for Model C1

**Figure S3** Photography of ionic porous polymers IPN-CSU23

**Figure S4** Possible routeline of IPN-CSUs

**Table S1** Elemental analysis (EA) for IPN-CSU22 and IPN-CSU23

**Figure S5** Thermo-gravimetric traces (TGA) for IPN-CSU2 and IPN-CSU23

**Figure S6** PXRD patterns of IPN-CSU22 and IPN-CSU23

**Figure S7** Scanning electron micrographs for IPN-CSU22(a, b) and IPN-CSU23(c,d)

**Figure S8** TEM for IPN-CSU22 with different magnification

**Figure S9** $CO_2$ adsorption/desorption curves of IPN-CSUs at 298K

**Figure S10** Possible reaction mechanism for $CO_2$ conversion by IPN-CSUs

**Table S2** $CO_2$ capacity and catalytic performance for model products

**Figure S11** NMR analysis for crude products from catalytic experiments

**Section S1. Materials**

Cyanuric chloride, and 1*H*-imidazole were purchased from Alfa Aesar Chemical Inc. and used as received without further purification. 1-Methyl-2-pyrrolidinone dichloromethane, acetone, tetrahydrofuran and chloroform were purified by distillation after removal of water over $CaH_2$ and stored with 4 Å molecular sieves prior to use. Unless other stated, all other solvents or regents used were commercially available and used without further purification.

**Section S2. Instruments and methods**

Using selected solvent mediums, hydrogen nuclear magnetic resonance ($^1H$ NMR) and carbon nuclear magnetic resonance ($^{13}C$ NMR) spectroscopic measurements of the soluble samples were recorded on a Bruker AV-400 spectrometer with tetramethylsilane (TMS) as the internal reference. Solid-state $^{13}C$ cross polarization magic angle spinning ($^{13}C$ CP/MAS) NMR spectra were obtained on a Bruker Avance III 400 NMR spectrometer. The spectra were obtained by using a contact time of 2.0 ms and a relaxation delay of 10.0 s. Using KBr disks, Fourier transform infrared spectroscopy (FT-IR) spectra of the powdered samples was collected in a transmission mode on a VARIAN 1000 FT-IR (scimitar series) spectrometer. Elemental analysis including carbon, hydrogen, and nitrogen was collected on a FlashEA 2000 Elemental Analyzer using a CHN model. Scanning electron microscopy (SEM) and Energy Dispersive X-ray spectroscopy (EDS) experiments were performed on a FEI Quanta-200 scanning electron microscope. For Transmission electron microscopy (TEM) measurements, samples were coated with a thin film of platinum. It was carried out on a JEOL JEM 3010 at 300 kV. Powder X-ray Diffraction (PXRD) pattern of the samples

was collected on a Bruker AXS D8 Discover multi-purpose high power X-ray diffractometer. Thermogravimetric analysis (TGA) was performed using a PERKIN ELMER TGA7 instrument, and the samples was investigated from 25 $^{o}C$ to 800 $^{o}C$ at a heating rate of 10 K min$^{-1}$ under $N_2$ atmosphere. The nitrogen adsorption and desorption isotherms were obtained by using ASAP 2020 volumetric adsorption analyzer at 77 K, and the surface area and pore size distribution of the sample were obtained using Bruner-Emmett-Teller (BET) and Barrett-Joyner-Halenda (BJH) models. The sample was degassed at 80 $^{o}C$ for 9 h under vacuum ($5*10^{-4}$ Pa) before the analysis. The cumulative apparent surface area for $N_2$ was calculated by using the BET model range from 0.025 to 0.10 bar for the sample. The pore size distribution of sample was calculated from the adsorption branches of the isotherms according to the Nonlocal Density Functional Theory (NLDFT) method by using a carbon slit pore model. The low-pressure nitrogen adsorption and desorption isotherms were measured at 273K. The adsorption and desorption isotherms of carbon dioxide ($CO_2$) were obtained from the ASAP 2020 volumetric adsorption analyzer at 273 K and 298 K up to 1 bar. Isosteric heat of adsorption ($Q_{ST}$) value of the samples towards nitrogen or $CO_2$ was calculated by using Clausius-Clapeyron equation on the data master offline data reduction software (Micrometrics). For the catalytic cycloaddition of $CO_2$ and epoxides, the reaction mixture after centrifugation to remove the IPN-CSUs catalyst was analysed by $^1H$ NMR spectroscopy to calculate the reaction yield.

**Section S3. AEC measurements of model products and IPNs**

The anion exchange capacity (AEC, chloride form) of model reaction products:

Model C1 (0.3 g) were dissolved in concentrated nitric acid and then 0.1M silver nitrate solution in water was added drowse to the clear solution. Finally, silver chloride as precipitate was isolated and washed by acetone, and dried under vacuum till constant weight. Yield: 0.15 g, corresponding to an AEC value of 3.4 mmol g-1.

AEC of IPF-CSUs: Typically, IPF-CSU-22 (0.3 g) was dissolved in concentrated nitric acid and the undissolved substance was filtered off to obtain a clear mixture solution. Secondly, 0.1M aqueous silver nitrate solution was added dropwise to the clear mixture solution in the condition of potassium chromate as indicator. Then, silver chloride as precipitate was isolated and washed by acetone, and finally dried under vacuum till constant weight.

**Section S4. Determination for isosteric heat of adsorption**

The adsorption enthalpies of these porous materials on $CO_2$ molecules were calculated by the adsorption isotherms at different temperatures (such as 273K and 298K) to study the binding ability of IPN-CSU adsorbent molecules to small gas molecules.It is clear that physical adsorption and chemical adsorption can be defined by the size of adsorption enthalpy. Combining the CO2 adsorption isotherms at 273K and 298K, the Clausius–Claperyron equation is fitted to calculate the adsorption enthalpy. The equation is as follows:

$$Q_{st} = R \times T_1 \times T_2 \,(\ln P_1/P_2)/(T_1\text{-}T_2) \qquad (1)$$

Qst is the adsorption enthalpy of CO2 by the porous body, R is the gas constant, and P1 and P2 are the corresponding CO2 adsorption capacity at T1=273K and T2=298K respectively.

**Section S4. Catalytic conversion of $CO_2$ to cyclic carbonates**

*Catalytic procedures*: A 3-neck round bottom flask was charged with IPN-CSUs catalyst (0.098 mmol), the epoxide 2-methyloxirane (12.5 mmol) with or without TBAB catalyst (1.44 mmol). Then, $CO_2$ (0.1 Mpa) was poured into the reactor as a reactant. The reaction mixture was stirred at 25 $^{o}$C for 48 h, after which the remaining

$CO_2$ was slowly evaporated. After centrifugation to remove the catalyst, the reaction mixture was analyzed by $^1$H NMR spectroscopy to determine the reaction yield.

## Section S4 Figures and tables

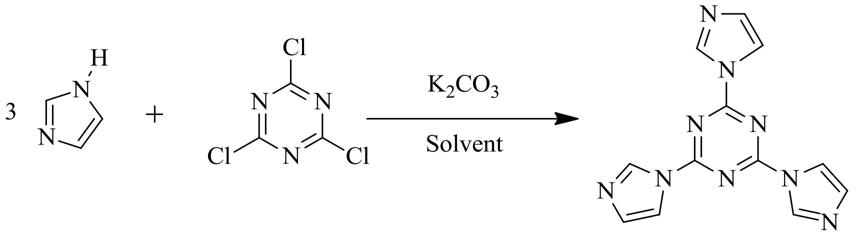


Model C1

**Scheme S1** Synthetic route of Model C1.

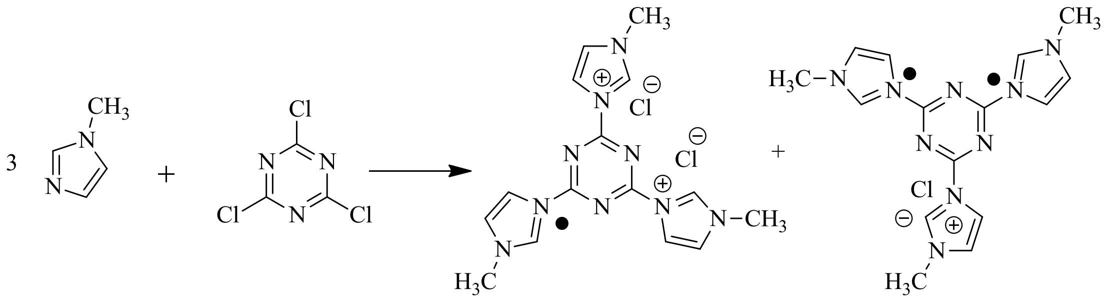


Model C2

Model C3

**Scheme S2** Synthetic route of Model C2 and Model C3.

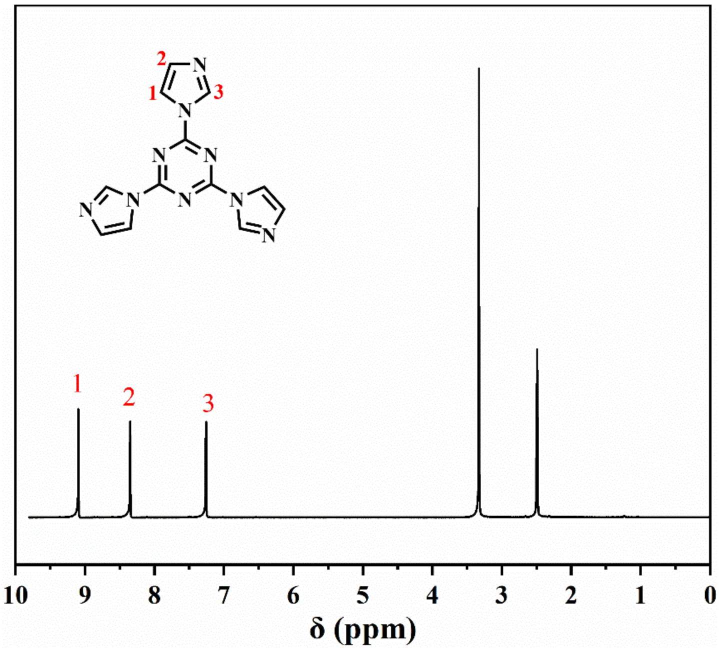


**Figure S1** $^{1}H$ NMR spectrum for Model C1.

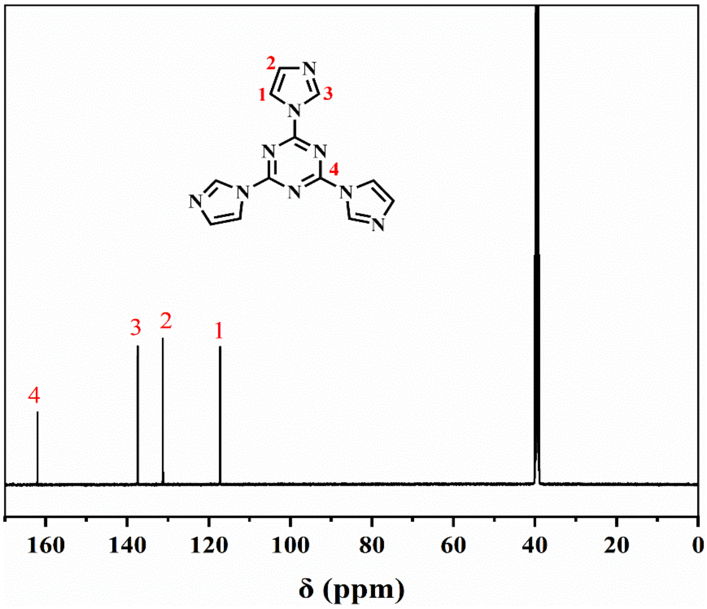


**Figure S2** $^{13}C$ NMR spectrum for Model C1.

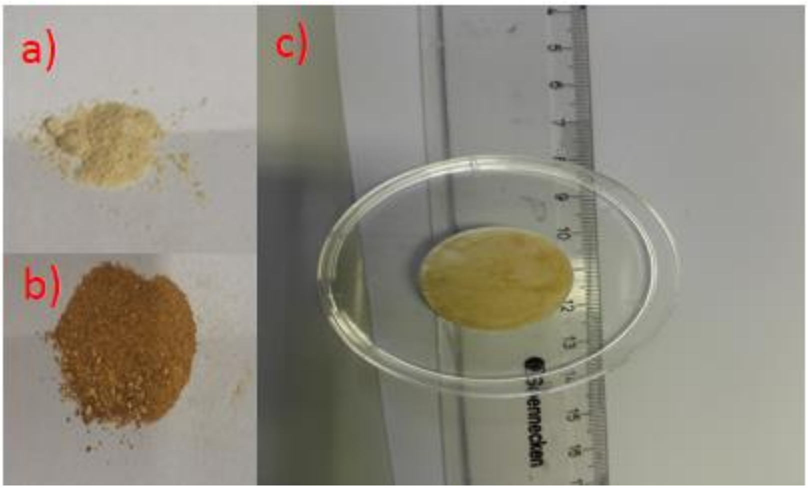


**Figure S3** Photography of ionic porous polymers IPN-CSU23.

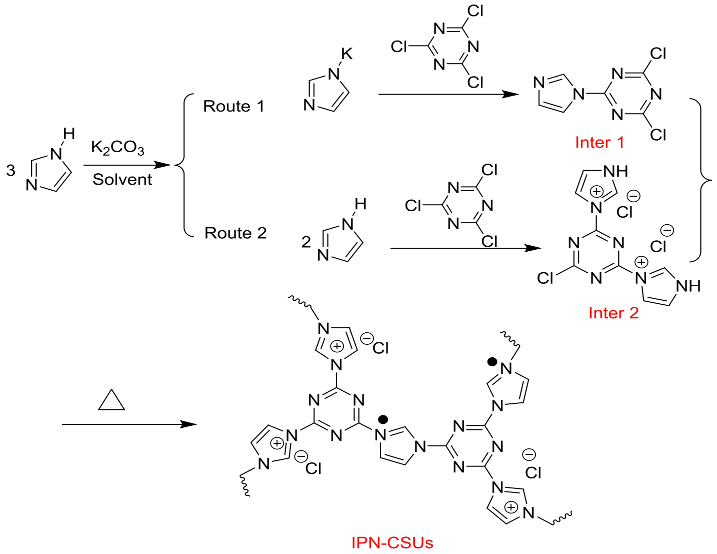


**Figure S4** Possible routeline of IPN-CSUs.

**Table S1** Elemental analysis (EA) for IPN-CSU22 and IPN-CSU23.

| Sample | EA theoretical value | | | EA found | | |
|---|---|---|---|---|---|---|
| | C | N | H | C | N | H |
| IPN-CSU22 | 32.41 | 33.59 | 2.42 | 33.42 | 33.37 | 2.28 |
| IPN-CSU23 | 32.41 | 33.59 | 2.42 | 33.08 | 33.01 | 2.09 |

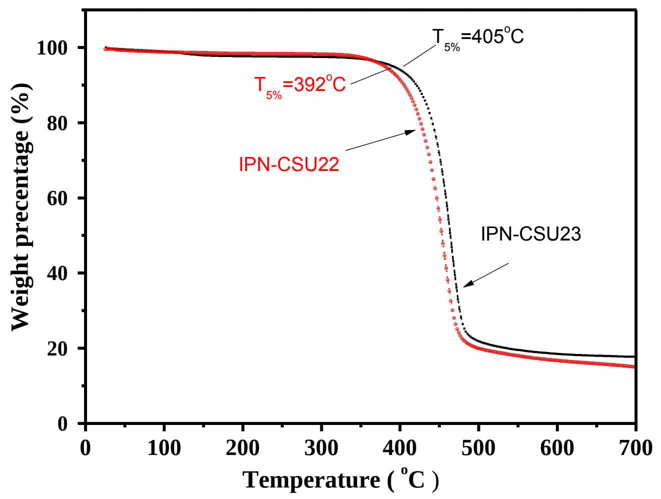


**Figure S5** Thermo-gravimetric traces (TGA) for IPN-CSU2 and IPN-CSU23.

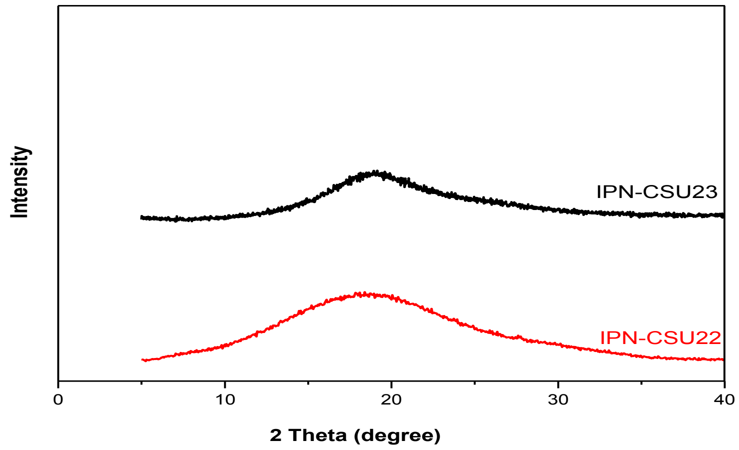


**Figure S6** PXRD patterns of IPN-CSU22 and IPN-CSU23.

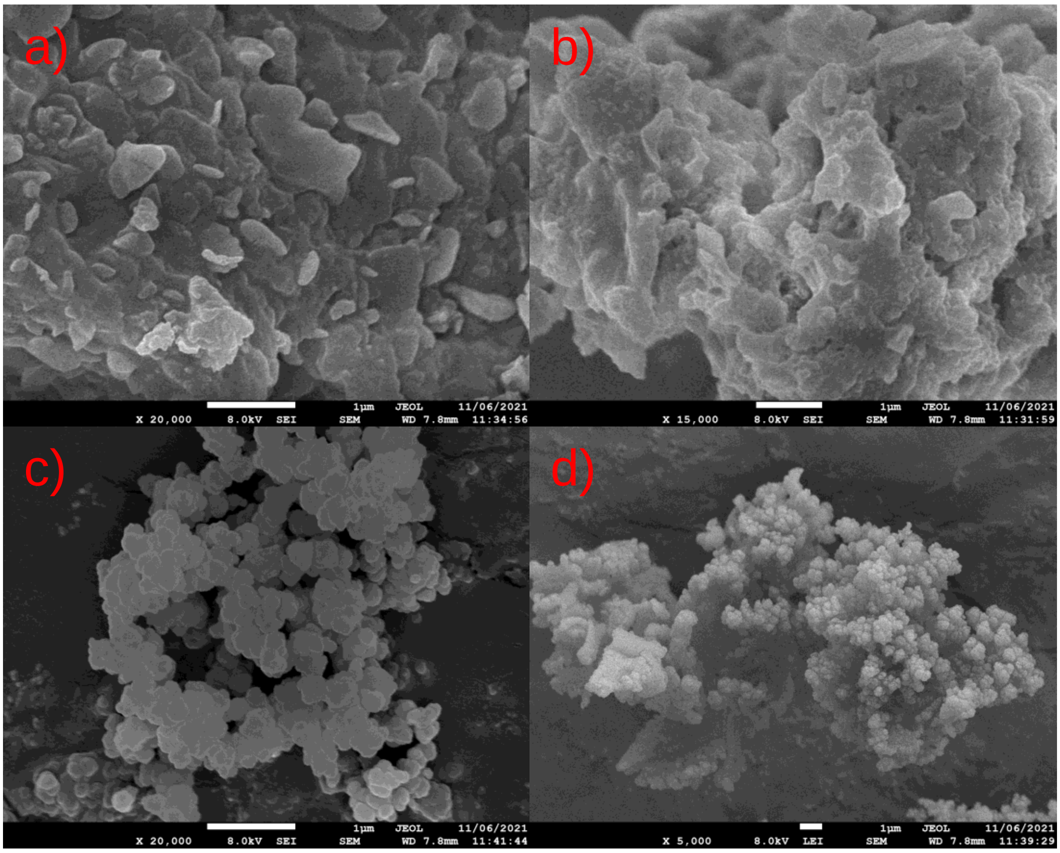


**Figure S7** SEM images for IPN-CSU22(a, b) and IPN-CSU23(c,d).

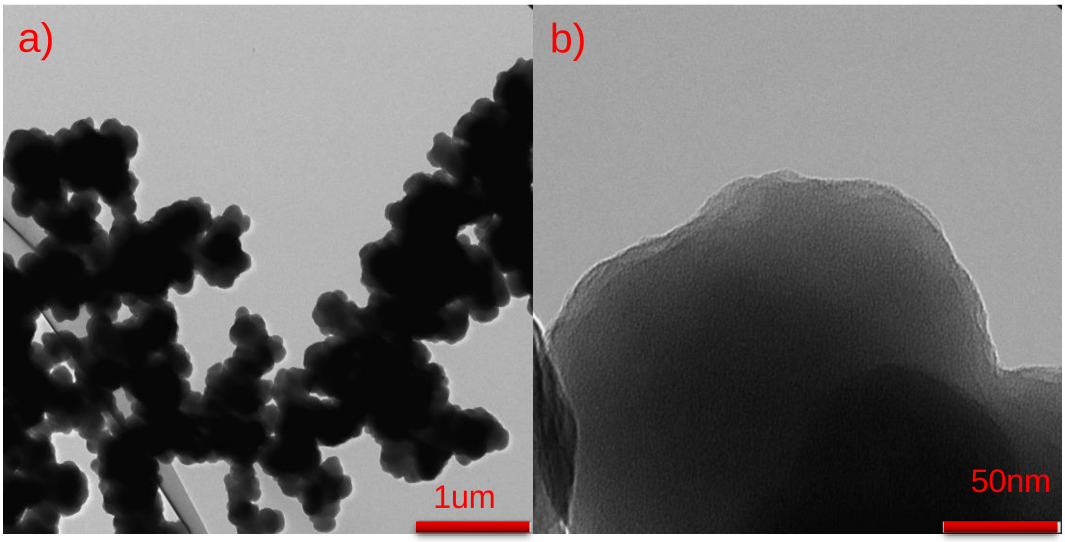


**Figure S8** TEM images for IPN-CSU22 with different magnification.

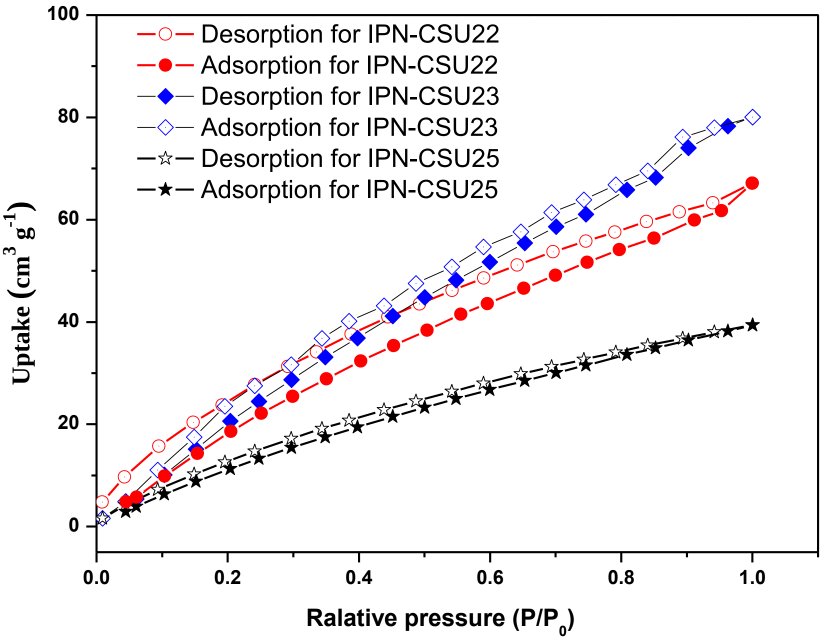


**Figure S9** $CO_2$ adsorption/desorption curves of IPN-CSUs at 273K.

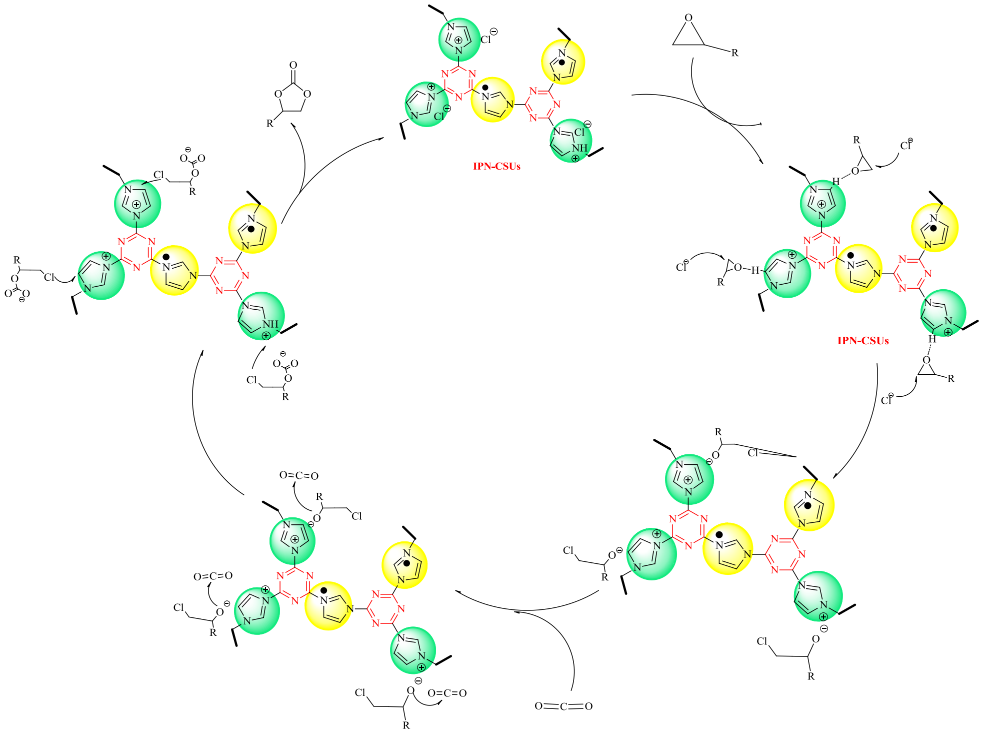


**Figure S10** Possible reaction mechanism for CO2 conversion by IPN-CSUs.

**Table S2** $CO_2$ capacity and catalytic performance for model products

| Sample | $CO_2$ capacity | | Catalytic fixation | | | |
|---|---|---|---|---|---|---|
| | $CO_2$ adsorption capacity[a] (cc/g) | Selectivity (v/v)[b] | Time (h) | Pressure (MPa) | Temp. ($^{o}$C) | Yield[c] (%) |
| Model C1 | 2.1 | 3.9 | 33.42 | 33.37 | 2.28 | -[d] |
| Model C2/C3 | 11.4 | 9.8 | 33.08 | 33.01 | 2.09 | 11.6 |

[a] 273 K and 1 bar. [b] Selectivity: initial ideal selectivity; [c] The yield of 4-methyl-1,3-dioxolan-2-one over model compound catalysts without the presence of TBAB (Determined by $^{1}$HNMR). [d] Not detected

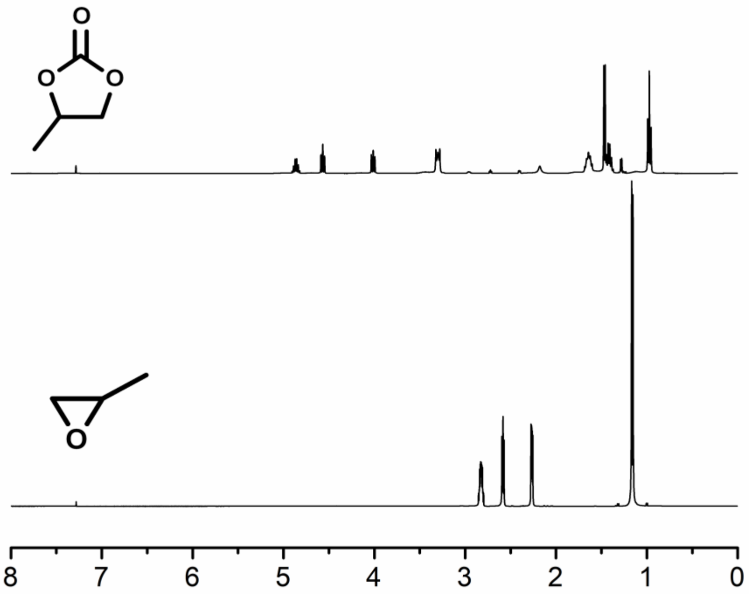


**Figure S11** $^{1}$H NMR (400 MHz, $CDCl_3$, 298 K) spectra of propylene oxide and its corresponding cyclic carbonate (crude sample) over IPN-CSUs/TBAB at 298K for 48 h.
